# Evaluation of Microstructure and Mechanical Properties of T6 Heat-Treated Al-Cu-Mg Aluminum Alloy Based on Laser Ultrasonics

**DOI:** 10.3390/ma19163423

**Published:** 2026-08-12

**Authors:** Chaochao Chen, Zhi Xu, Anmin Yin

**Affiliations:** 1 Beijing University of Science and Technology Design and Research Institute Co., Ltd., Beijing 100083, China; ustbccc@163.com; 2 Institute of Engineering Technology, University of Science and Technology Beijing, Beijing 100083, China; 3 College of Mechanical and Automotive Engineering, Ningbo University of Technology, Ningbo 315336, China; xuzhi@nbut.edu.cn; 4 School of Mechanical Engineering and Intelligent Manufacturing, Ningbo University, Ningbo 315211, China

**Keywords:** laser ultrasonic, aluminum alloy, microstructure, mechanical properties, nondestructive evaluation

## Abstract

At present, the detection methods for the microstructure and mechanical properties of aluminum alloys are mainly based on SEM, EBSD, TEM, tensile tests, and microhardness tests, which are time-consuming and destructive. In this paper, laser ultrasonic non-destructive detection is employed to obtain ultrasonic signals from Al-Cu-Mg aluminum alloy subjected to various heat treatment processes. The results reveal empirical correlations between the characteristic values of the ultrasonic signals and the material’s state. Specifically, the characteristic values exhibit an inverse correlation with the precipitated phase content. When both the precipitated phase content and the average grain size vary significantly, distinct deviations in the characteristic values are observed, which can serve as indicators of microstructural changes. The extracted ultrasonic eigenvalues also show promising, empirically derived correlations with mechanical properties, with frequency-domain attenuation coefficients demonstrating relatively higher sensitivity based on fitting analyses within the current dataset. These observed variations are tentatively discussed as plausible consequences of grain boundary scattering and changes in matrix solid solution strengthening associated with precipitate dissolution. Overall, the findings suggest the potential of laser ultrasonics as a rapid non-destructive evaluation tool, providing a preliminary scientific basis for further development of methods to assess the microstructure and mechanical properties of Al-Cu-Mg alloys within the tested parameter space.

## 1. Introduction

Al-Cu-Mg alloy, as a high-strength aluminum alloy in aluminum alloys, has high tensile strength and fatigue strength, and is widely used in various fields because of its excellent properties [1,2], especially in aviation. The increase in Mg and Cu can significantly promote the formation and strength improvement in the θ phase of Al-Cu-Mg series aluminum alloy [3,4]. However, there will be a large amount of precipitated phases, and the existence of a precipitated phase will have a very important impact on the performance of the metal [5]. The microstructure detection methods mainly include Scanning Electron Microscopy (SEM), Electron Backscattered Diffraction (EBSD) detection, etc. The main testing methods for mechanical properties are tensile test, microhardness test and so on. Although these methods can obtain accurate results, they are destructive and time-consuming, and cannot characterize the microstructure and mechanical properties of every part of the workpiece.

Ultrasonic non-destructive evaluation offers a rapid and comprehensive approach for characterizing the microstructure and mechanical properties of metallic materials without causing damage. Numerous studies [6,7] have demonstrated the feasibility of using ultrasonic methods for material characterization. Laser ultrasonic technology, as an advanced non-destructive technique developed in recent years, possesses distinct advantages including non-contact operation, long-distance testing capability, wide detection range, high speed, and high precision [8,9].

In the field of microstructure inspection, laser ultrasonic techniques have been applied to grain size evaluation. Xue and Dong et al. [10,11,12] used laser ultrasonic experiments combined with COMSOL finite element simulation to detect and predict average grain size. Garcin et al. [13,14,15] evaluated grain sizes in Inconel 718, pure titanium, and Ti-6Al-4V using in situ laser ultrasonic technology. Sundin, Rodrigues et al. [16] found a linear relationship between ultrasonic P-wave velocity and α-phase content measured by SEM. In terms of precipitate characterization, Zhu et al. [17] established a relationship between precipitated phase size and ultrasonic attenuation coefficient in 7055 aluminum alloy, demonstrating that ultrasonic attenuation is related to precipitate size. For mechanical property evaluation, Sano et al. [18] measured the material properties of steel sheets using laser ultrasonics and found good agreement with SEM-EBSD and tensile test results. Popovich et al. [19,20] demonstrated the feasibility of measuring structural heterogeneity in layered metal materials through ultrasonic velocity measurements. Zinin et al. [21] achieved measurement errors of less than 2% for sample thickness, P-wave velocity, and S-wave velocity using LU-DAC technology. Zhan et al. [22] studied residual stress in laser additive manufactured titanium alloy and established an empirical formula for residual stress assessment in LAM TC4. More recently, Xu et al. [23] found a good correlation between laser ultrasonic eigenvalues and the yield strength and microhardness of aluminum alloys.

Despite these advances, several important gaps remain. Previous studies have largely focused on single-factor effects—either grain size or precipitated phase content—in specific alloy systems such as 7055 aluminum alloy or nickel-based superalloys. Systematic investigations on the coupled effects of both grain size and precipitate content on laser ultrasonic characteristic values in Al-Cu-Mg aluminum alloys are still limited. Moreover, the quantitative relationships between these microstructural features and ultrasonic parameters in this specific alloy system have not been fully established. This lack of comprehensive understanding restricts the practical application of laser ultrasonics for rapid, non-destructive evaluation of Al-Cu-Mg alloys.

To address these gaps, the present work systematically investigates the coupled influence of grain size and precipitated phase content on laser ultrasonic responses in Al-Cu-Mg aluminum alloy subjected to various heat treatment conditions. Unlike previous studies that primarily examined single-factor effects, this study explicitly analyzes how simultaneous variations in both grain size and precipitate content affect ultrasonic characteristic values. Furthermore, empirical relationships between ultrasonic characteristic values and key mechanical properties—including tensile strength, yield strength, and microhardness—are established and evaluated based on a limited set of 14 heat-treated specimens (A0–A13). Through this comprehensive analysis, we propose a preliminary evaluation framework for assessing the microstructure and mechanical properties of Al-Cu-Mg aluminum alloy, providing a non-contact, rapid, and non-destructive approach for material characterization within the investigated microstructural and processing range. It should be emphasized that the proposed empirical correlations and reference intervals are derived from the current dataset (specimens A0–A12, with sample A13 excluded from the correlation analysis due to its significant deviation from the general trend) and therefore require further independent validation before they can be generalized to other alloy systems or processing conditions.

## 2. Experimental Materials and Methods

### 2.1. Experimental Materials

The material investigated in this study is a rolled Al-Cu-Mg aluminum alloy sheet with a nominal thickness of 2 mm. The alloy was supplied in the rolled condition by the manufacturer; its chemical composition, as provided by the supplier, is listed in Table 1. The as-received material was subsequently cut into rectangular specimens of 55 × 50 mm for heat treatment and further characterization.

#### 2.1.1. Heat Treatment Procedures

To obtain a range of microstructures and mechanical properties, the specimens were subjected to annealing and T6 solution-aging treatments using an HX4A-15 thermoelectric furnace (Zhongda Qiang, Dongguan, China), with a temperature control accuracy of ±2 °C. The detailed heat treatment schedules are presented in Table 2. The annealing treatments were performed at temperatures ranging from 200 to 500 °C with varying holding times, followed by either furnace cooling (FC) or water quenching (WC). For the T6 treatment, the solutionized specimens were aged at 180 °C for 720 min and subsequently air-cooled (AC). These heat treatment conditions were selected to systematically alter the grain size and precipitate content, which in turn influence the tensile properties and microhardness of the alloy.

#### 2.1.2. Microstructural Characterization

For microstructural observation, the heat-treated specimens were sectioned, mounted, and ground using a series of SiC abrasive papers from 200 to 5000 grit. Following grinding, the samples were polished with diamond suspensions of 2.5, 1, and 0.5 μm on a silk cloth to obtain a mirror-like surface. After each polishing step, the specimens were ultrasonically cleaned in anhydrous ethanol for 3 min and dried with cold air.

To reveal the precipitated phases, the polished samples were etched with Keller’s reagent (1 mL HF, 1.5 mL HCl, 2.5 mL HNO_3_, and 95 mL H_2_O) for 18 s. The precipitate morphology and composition were examined using a Hitachi SU5000 field emission scanning electron microscope (SEM) equipped with an energy-dispersive X-ray spectroscopy (EDS) system. The accelerating voltage was set to 15 kV, and the working distance was maintained at approximately 10 mm. The precipitate area fraction was quantified using ImageJ 1.54h software.

For electron backscatter diffraction (EBSD) analysis, the specimens were further ground up to 5000 grit and then electropolished in a solution of 10 vol.% HClO_4_ and 90 vol.% C_2_H_5_OH at 20 V for 20 s. EBSD scans were performed using the same Hitachi SU5000 SEM equipped with an Oxford Instruments EBSD detector, operating at an accelerating voltage of 20 kV. A step size of 1 μm was used, and the data were post-processed using HKL Channel 5 software to determine the average grain size, with a minimum of 200 grains analyzed for each condition.

#### 2.1.3. Mechanical Property Testing

Tensile tests were conducted on an MTS810 electro-hydraulic servo universal testing machine following the Chinese standard GB/T 228.1-2021 [24]. Dog-bone-shaped specimens with a gauge length of 50 mm were prepared from each heat-treated condition. To ensure reproducibility, three tensile specimens were tested for each condition, and the average values of tensile strength, yield strength (0.2% offset), and elongation are reported.

Vickers microhardness measurements were performed using a HXD-1000TMC/LCD microhardness tester with a load of 100 g and a dwell time of 15 s. For each specimen, 10 indentations were made on the polished surface, and the average hardness value was calculated.

### 2.2. Laser Ultrasonic Detection Method

**Figure 1:**
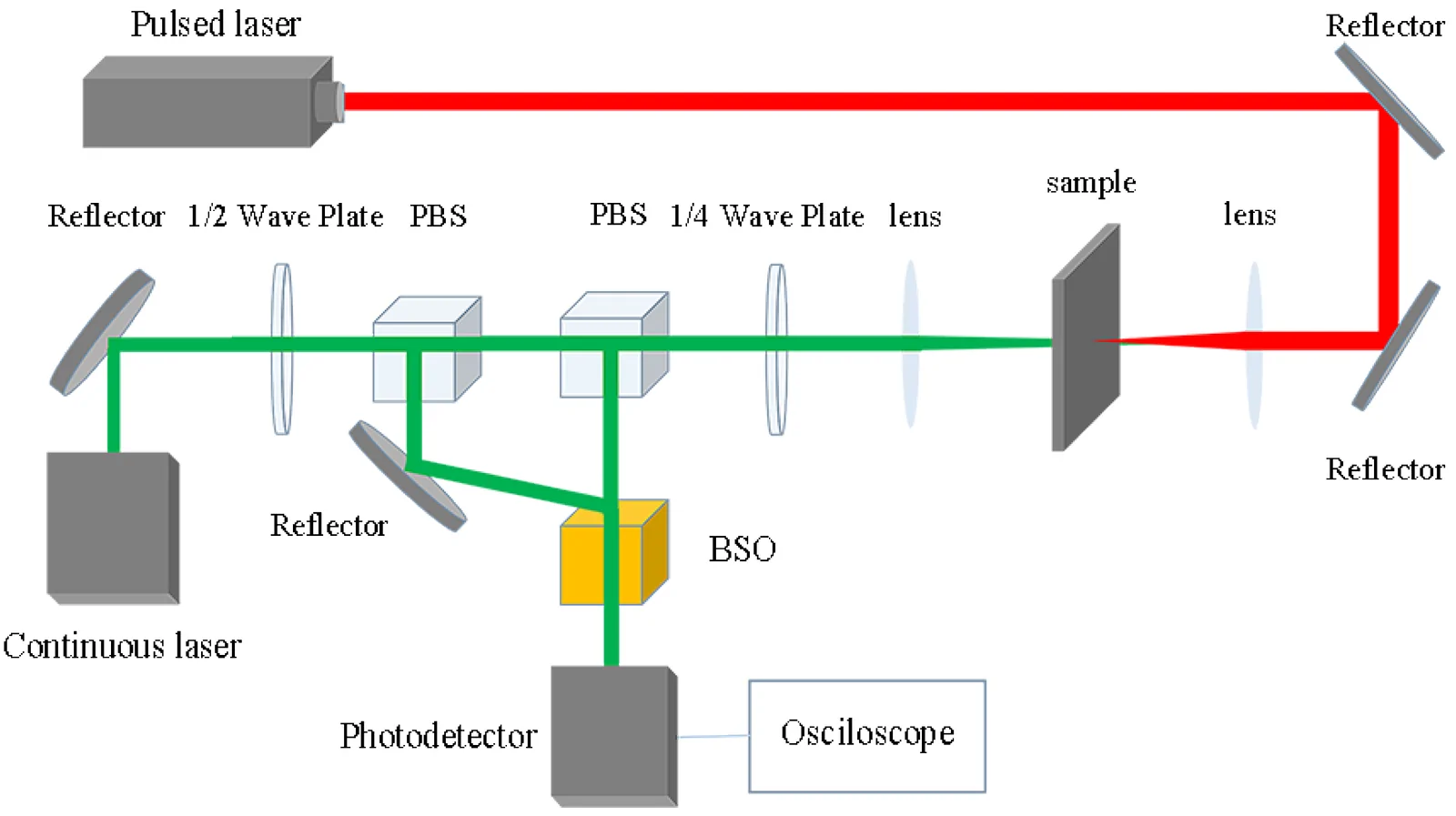
Laser ultrasonic detection system.

The laser ultrasonic experimental system employed in this study is illustrated in Figure 1, which consists of three main subsystems: a laser excitation source, an ultrasonic detection system, and a signal acquisition unit. For ultrasonic generation, a Dawa-series Q-switched Nd: YAG pulsed laser was used, which offers a low threshold and adjustable output power levels suitable for this experiment. The pulsed laser was operated at a wavelength of 1064 nm, with a pulse width of 8 ns and a single-pulse energy of 500 mJ. The pulse repetition rate was set to 10 Hz, and the laser beam had a diameter of approximately 6 mm with a divergence angle of 0.001 rad. The emitted pulse laser (indicated by the red beam path in Figure 1) was directed through a reflector and then focused onto the sample surface using a convex lens. This localized heating induced transient thermal expansion, thereby generating ultrasonic waves within the specimen via the thermoelastic mechanism.

For non-contact detection of the generated ultrasonic waves, a two-wave mixing interferometer based on a photorefractive Bi_12_SiO_20_ (BSO) crystal was employed on the opposite side of the sample. This detection system operates on the principle of dynamic holographic interferometry and primarily comprises a continuous-wave (CW) laser, the interferometer assembly, the BSO crystal, and a polarization beam splitter (PBS). The CW detection laser (indicated by the green beam path in Figure 1) was set to a wavelength of 532 nm, with an output power of 300 mW, a beam diameter of 1.5 mm, a linewidth of less than 1 × 10^−14^ m, and a divergence angle of 0.0015 rad. The CW laser beam was split by the PBS into a signal beam and a reference beam. The signal beam was directed onto the sample surface via the interferometer optics, and the diffusely reflected light from the surface was collected. When the pulsed laser excited the sample, the resulting surface displacement modulated the phase and intensity of the reflected signal beam. This modulated signal beam, carrying the ultrasonic displacement information, was then combined with the internal reference beam inside the BSO photorefractive crystal, where they interfered to form a dynamic holographic grating. Through this two-wave mixing process, a portion of the reference beam was effectively transferred to the signal beam, amplifying the intensity variations caused by the surface motion. The modulated optical signal was finally converted into an electrical signal by a photodetector and subsequently recorded by a digital oscilloscope.

The experiments were performed in a transmission-mode configuration, where the excitation laser and the detection laser were positioned on opposite sides of the sample, as shown in Figure 1. The pulsed excitation beam was focused on the front surface, while the CW detection beam was focused on the back surface at a location directly opposite to the excitation spot to maximize signal amplitude. The oscilloscope was configured with a sampling rate of 1250 MHz and a bandwidth of 500 MHz. To improve the signal-to-noise ratio, each recorded waveform was obtained by averaging eight consecutive acquisitions, which was performed through the oscilloscope’s hardware averaging function. For each sample, measurements were taken at three different positions to account for potential local variations, and the reported results represent the average values across these positions. The entire setup was mounted on a two-dimensional translation stage, allowing precise positioning of the sample relative to the laser beams.

## 3. Experimental Results and Analysis

### 3.1. Laser Ultrasonic Signal Feature Extraction

Laser ultrasonic experimental signals contain significant noise components that require effective denoising prior to further analysis and feature extraction. The application of appropriate denoising methods can substantially improve the detection accuracy of laser ultrasonic systems and is therefore a critical step for practical implementation. Commonly used signal processing techniques include Wavelet Threshold Denoising (WT) [25] and Empirical Mode Decomposition (EMD) [26]. In this study, variational mode decomposition (VMD) is employed to process the laser ultrasonic signals.

**Figure 2:**
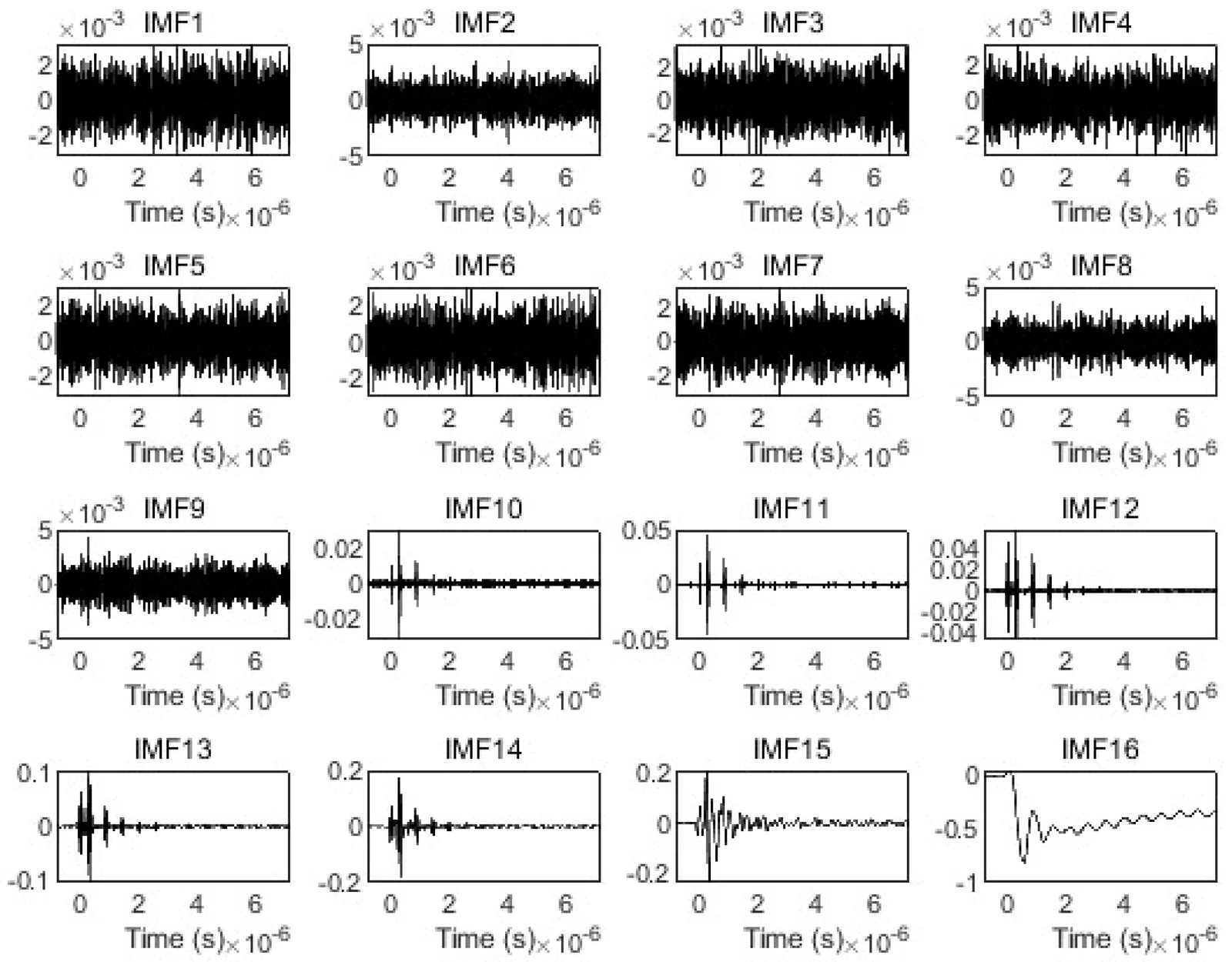
Results of variational mode decomposition.

The VMD method, originally proposed by Dragomiretskiy in 2014 [27], treats any signal as a combination of modal functions with distinct bandwidths. This approach converts the signal processing problem into a variational model optimization task, where the center frequency and bandwidth of each mode are determined through iterative search for the variational model extrema, thereby achieving decomposition of noisy signals [28]. In the present work, VMD analysis is performed using MATLAB R2023a software with the VMD function and FFT function. The decomposition parameters are set as follows: the quadratic penalty factor α is 2000, which effectively prevents modal aliasing in most signals. The number of decomposition modes K is determined using the normalized center frequency observation method [29]. Since the intrinsic mode functions are defined by their center frequencies, and different intrinsic mode functions should possess distinct center frequencies, the occurrence of identical center frequencies between two different modes indicates over-decomposition. For the Al-Cu-Mg aluminum alloy laser ultrasonic signals, K is set to 16, yielding 16 finite-bandwidth modal functions, as shown in Figure 2.

**Figure 3:**
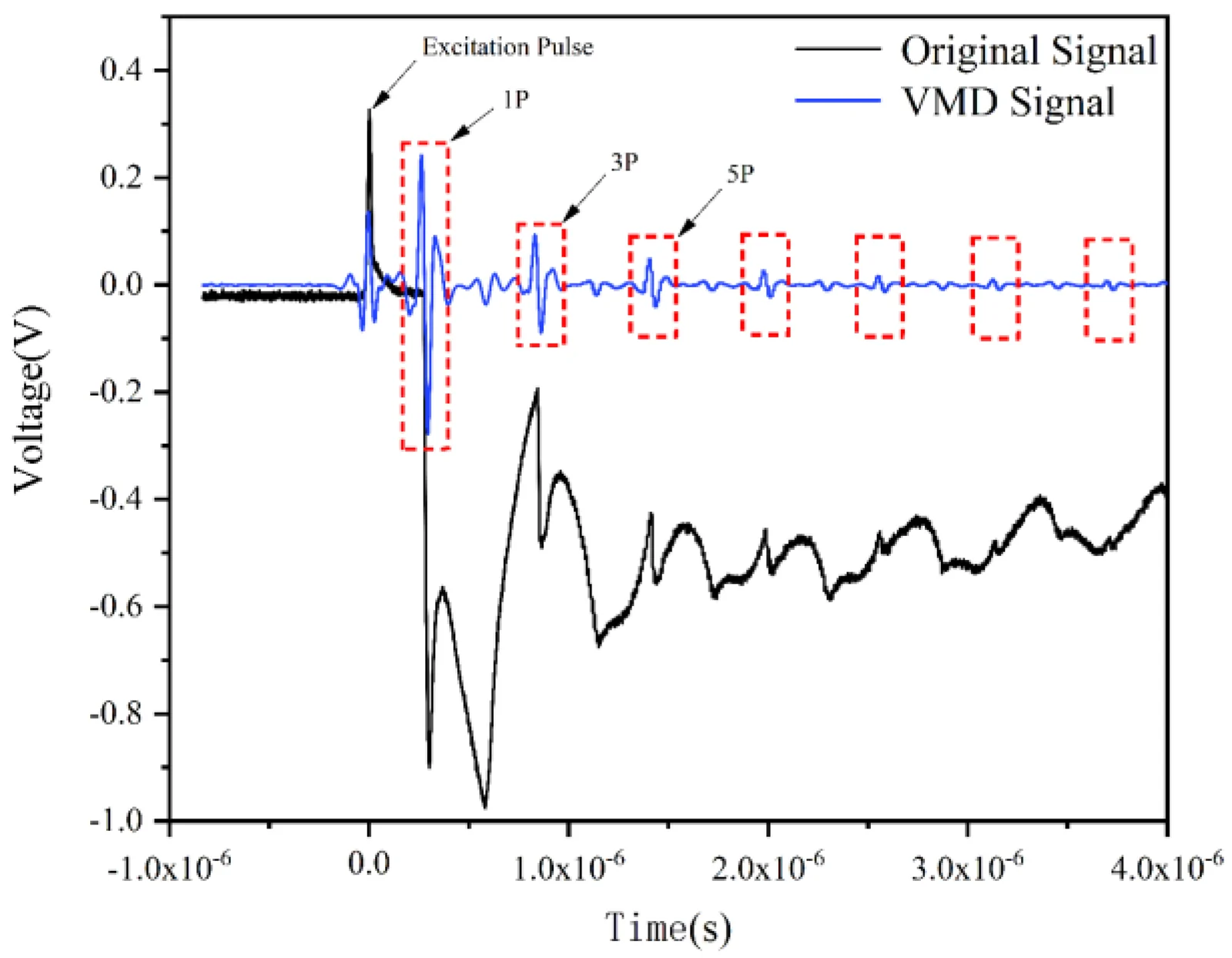
Original signal and VMD reconstruction signal.

As observed from the decomposition results, IMF1 through IMF9 exhibit pronounced high-frequency noise characteristics. IMF15 and IMF16 show very low frequencies with near-zero peak amplitudes, which correspond to baseline drift caused by thermal expansion. Since the longitudinal ultrasonic wave signal possesses a specific structural pattern, IMF10, IMF11, IMF12, IMF13, and IMF14 are selected for signal reconstruction. The reconstructed laser ultrasonic signal is shown as the blue curve in Figure 3. Following the removal of high-frequency noise and baseline drift, the signal exhibits improved smoothness with clearly identifiable echo peaks of the longitudinal wave, facilitating subsequent feature extraction and calculation.

The laser ultrasonic laboratory detection system employs a pitch-catch configuration for longitudinal wave reception. When the longitudinal wave signal propagates from the sample surface to the bottom for the first time, it is received by the dual-wave mixed interferometer, corresponding to the excitation pulse (indicated as 1P in Figure 3). The longitudinal wave is then reflected back to the excited surface by the bottom, re-reflected to the bottom, and received again. This process repeats, with the ultrasonic propagation distance between two consecutive echoes being twice the sample thickness. The thickness L of each specimen is measured using a micrometer screw gauge at three different positions, and the average value is recorded. The average thicknesses of the specimens are presented in Table 3.

**Figure 4:**
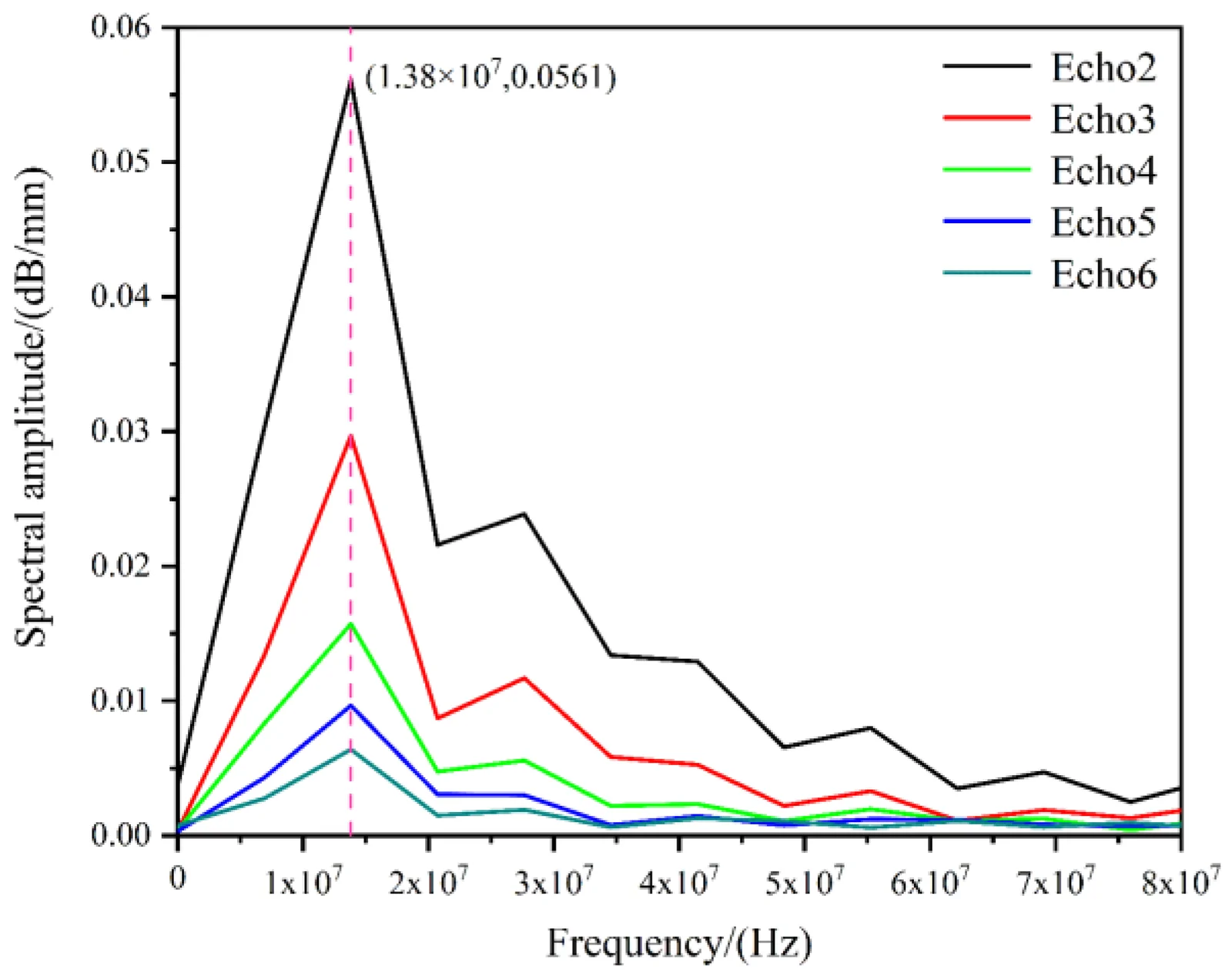
Ultrasonic p-wave echo spectrum.

The attenuation of ultrasonic waves propagating in a material primarily consists of absorption attenuation and scattering attenuation. Microstructural variations predominantly affect the scattering attenuation component, enabling the characterization of material microstructure through calculation of the ultrasonic attenuation coefficient. In this study, the amplitude method is employed to calculate the attenuation coefficient by extracting peak amplitudes from the denoised echo signals. Since near-field scattering effects affect the first bottom echo (1P), the attenuation coefficient is calculated using the second echo (3P) and third echo (5P) in Figure 2, as expressed in Equation (1):

(1) $$\alpha =\frac{1}{2L}\mathrm{lg}(\frac{E_{1}}{E_{2}})$$

where E1 and E2 are the peak amplitudes of echoes 3P and 5P, respectively, and L is the sample thickness. Correspondingly, the ultrasonic velocity v is determined by the time-of-flight between these successive echoes according to Equation (2):

(2) $$v=\frac{2L}{t_{2}-t_{1}}$$

where t1 and t2 are the arrival times of echoes 3P and 5P. To analyze the frequency characteristics, five complete echo signals (from the second to the sixth echoes) were intercepted from the VMD-denoised signal in Figure 3 and transformed via fast Fourier transform, yielding the spectrograms shown in Figure 4.

**Figure 5:**
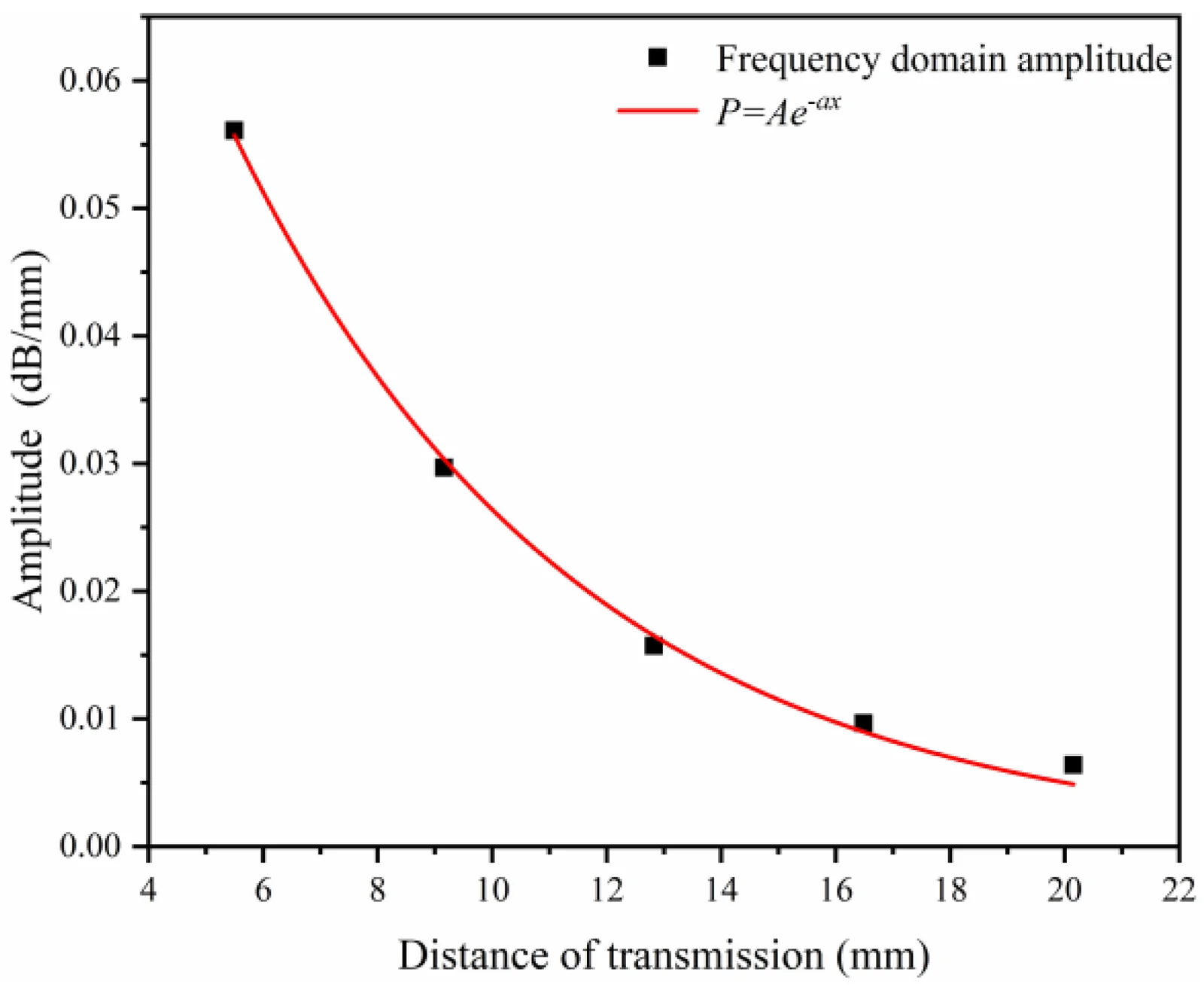
Frequency-domain attenuation coefficient fitting results.

The frequency of 13.8MHz corresponds to the central frequency of the received ultrasonic signal, which is determined by the laser excitation source and the transducer response characteristics of the interferometer. This frequency exhibits the highest signal-to-noise ratio and is therefore selected for amplitude extraction. The amplitude between adjacent echoes is substituted into Formula (2) to calculate the attenuation coefficient in the frequency domain. The frequency-domain attenuation coefficients between neighboring echoes are calculated by coupling with Formula (3), and the coupling results are shown in Figure 5.

(3) $$P=Ae^{-\alpha_{Frequency}x}$$

where *P* is the amplitude after the Fast Fourier transform, *α* is the attenuation coefficient in the frequency domain and *x* is the ultrasonic propagation distance.

After coupling the frequency-domain attenuation coefficients between different echoes, the corresponding ultrasonic propagation distances and amplitudes exhibit a high goodness of fit, with the coefficient of determination (R^2^) exceeding 0.95 for all fittings. The laser ultrasonic characteristic values calculated for each sample are shown in Table 4.

### 3.2. Relationship Between Laser Ultrasonic Characteristic Values and Microstructural Features

#### 3.2.1. Laser Ultrasonic Detection of Grain Size

**Figure 6:**
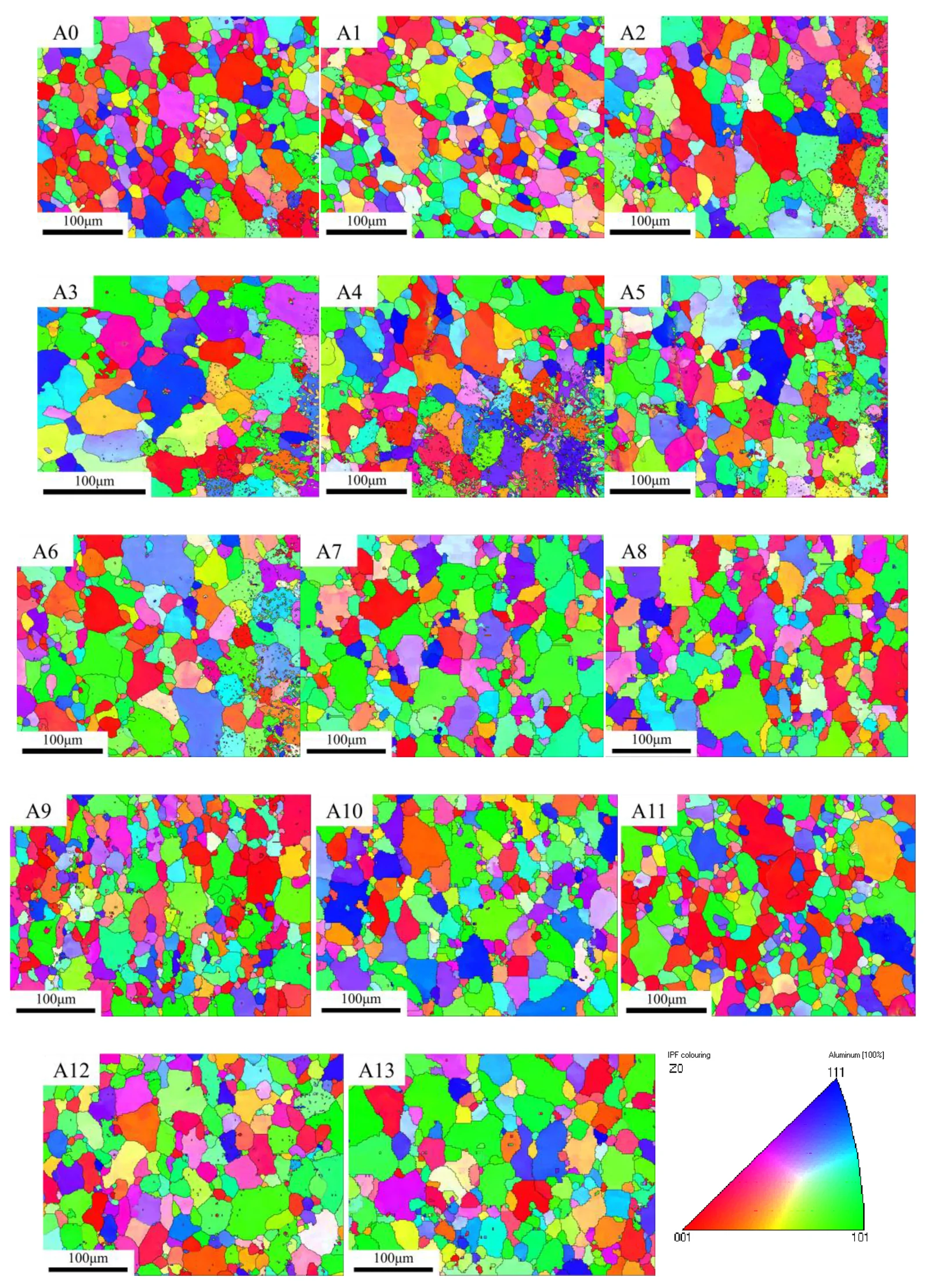
EBSD orientation diagram of Al-Cu-Mg aluminum alloy sample.

EBSD data were acquired using a Hitachi SU5000 SEM equipped with an Oxford Instruments EBSD detector, operated at 20kV with a step size of 1 μm. Post-processing was performed using HKL Channel 5 software. The raw orientation maps were first cleaned using the automatic noise reduction routine to remove non-indexed points. Grain boundaries were defined as misorientations greater than 10° to exclude subgrain structures and second-phase particles. At least 200 grains were analyzed for each condition to obtain reliable average grain size statistics. The orientation diagram of the sample after noise reduction is obtained, as shown in Figure 6. The average grain size D of the Al-Cu-Mg aluminum alloy sample is calculated by using the grain statistical method provided in HKL Channel 5 software. The results are shown in Table 5.

The standard deviation σ of the log–normal distribution reflects the uniformity of grain size in the specimen. A smaller σ indicates a more uniform grain size distribution. As shown in Table 6, the standard deviation of most specimens is approximately 0.75. Among them, specimen A1 exhibits the smallest standard deviation of 0.62, indicating the most uniform grain size distribution. Specimens A3, A6, and A12 show relatively larger standard deviations, suggesting poorer uniformity in grain size distribution, with specimen A3 displaying the most non-uniform grain size distribution, characterized by a standard deviation of 0.90.

Additionally, the grain size distribution data for each specimen were extracted from the EBSD MAP files using HKL Channel 5 software. These data were then imported into MATLAB, where a logarithmic transformation was performed to calculate the standard deviation (σ) and the expected value (μ) of the log–normal distribution. By substituting σ and μ into the Turner grain size model, the Turner grain size ($\tilde{D}$) was obtained for each specimen, and the corresponding results are presented in Table 6 alongside the average grain sizes.

The average grain size of the Al-Cu-Mg alloy ranges from approximately 10.6 to 17.0 μm (see Table 5), while the central frequency of the laser-generated ultrasonic wave is about 13.8 MHz, corresponding to a wavelength of roughly 0.45 mm in aluminum. This grain-size-to-wavelength ratio nominally satisfies the Rayleigh scattering condition. However, the alloy contains substantial amounts of Al_2_Cu and Al_2_CuMg precipitates (Table 7), which act as additional scatterers with acoustic properties distinct from the aluminum matrix. The total ultrasonic attenuation therefore arises from the combined effect of grain boundary and precipitate scattering, and the precipitate volume fraction varies with heat treatment conditions in a manner that correlates with grain size evolution.

**Figure 7:**
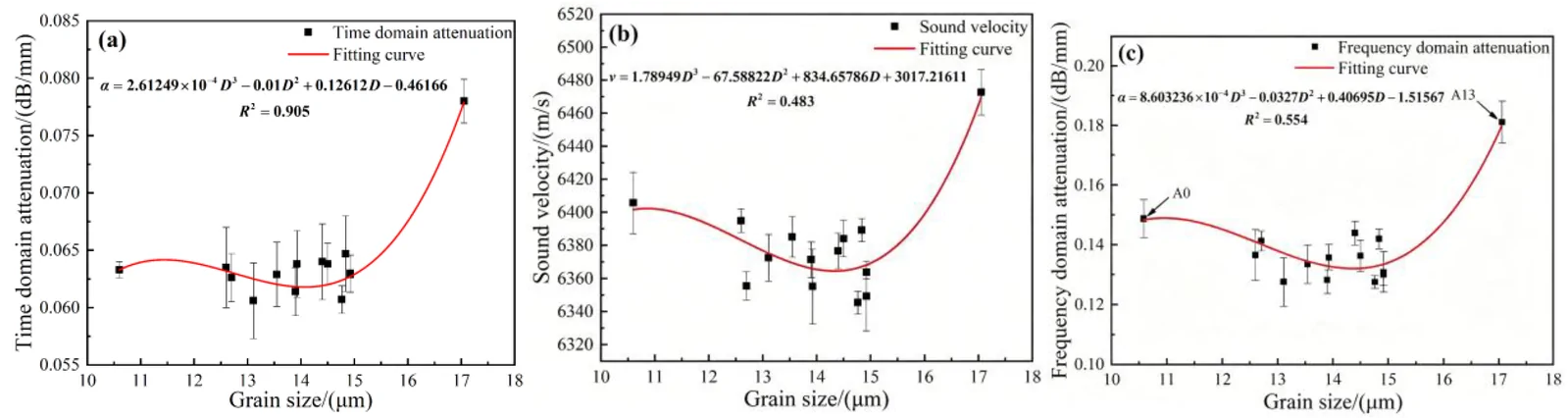
Coupling curves of average grain size with (**a**) time-domain attenuation coefficient, (**b**) sound velocity, (**c**) frequency-domain attenuation coefficient. Error bars represent the standard deviation of three repeated ultrasonic measurements at different positions on each specimen.

In this multi-phase system, the conventional Rayleigh law—which assumes a single type of scatterer—cannot adequately describe the attenuation behavior, because the precipitates act as secondary scatterers with acoustic properties distinct from the aluminum matrix, and their volume fraction varies with heat treatment in a manner coupled to grain size evolution. Consequently, an empirical second-order polynomial fitting is adopted to capture the coupled dependence of attenuation on both grain size and precipitate content. This polynomial form was chosen because the experimental data exhibited a monotonic but slightly curved trend against grain size, indicating that a linear model would be insufficient; higher-order terms were avoided to prevent overfitting given the limited number of data points. The fitting parameters are treated as empirical coefficients that describe the effective scattering efficiency within the investigated microstructural range, rather than as physically meaningful constants; their numerical values are dataset-dependent and should not be extrapolated beyond the examined conditions. The fitting yields a coefficient of determination (R^2^) of 0.91 (Figure 7a).

Similarly, the grain size was fitted against ultrasonic velocity and frequency-domain attenuation coefficient, but the correlations were considerably weaker, with R^2^ values of 0.48 and 0.55, respectively (Figure 7b,c). These weak correlations indicate that the latter two ultrasonic parameters cannot serve as reliable indicators for grain size estimation in this alloy system.

#### 3.2.2. Laser Ultrasonic Detection of Precipitated Phase

**Figure 8:**
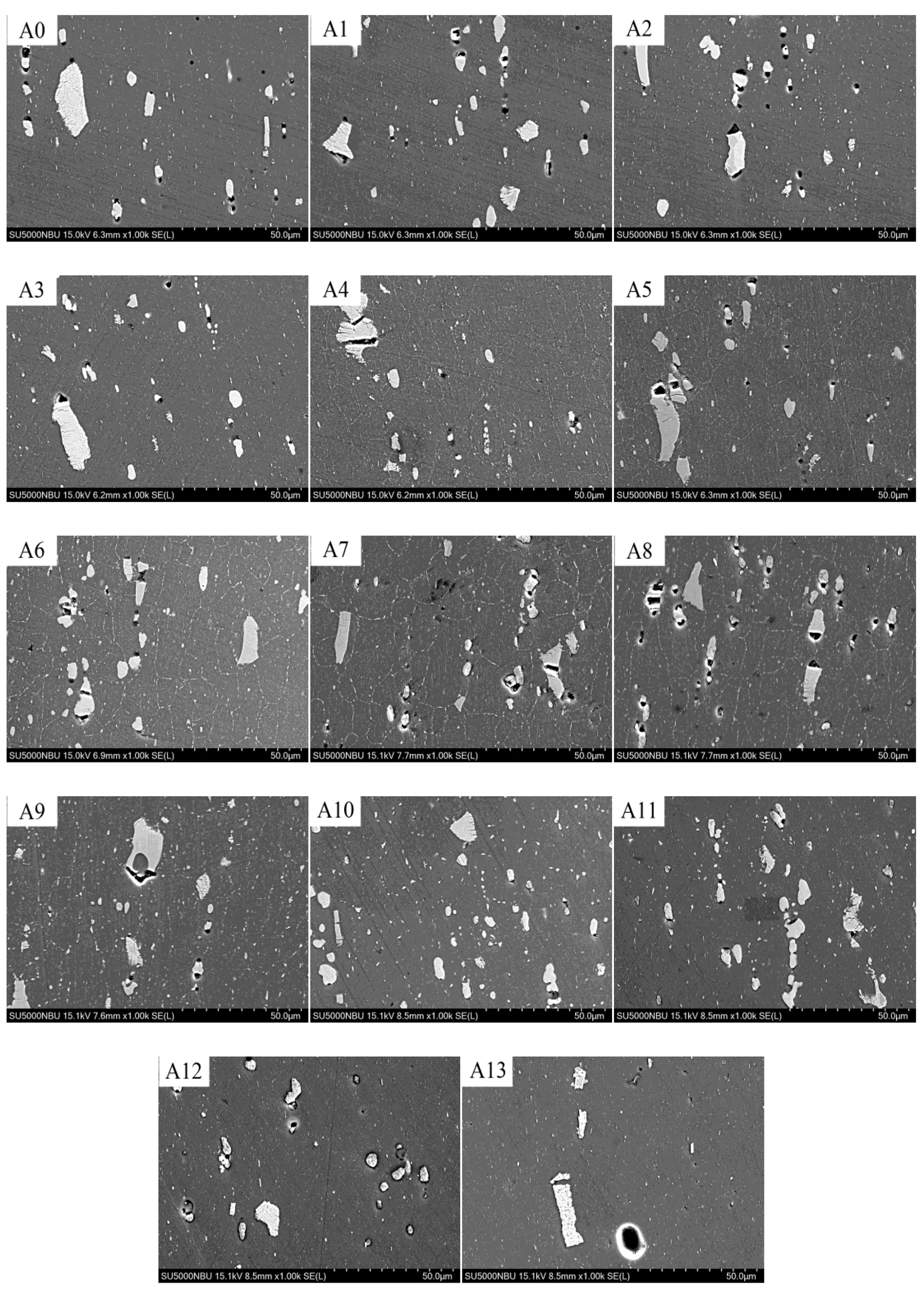
Precipitated phase measured by SEM.

Through metallographic experiments, SEM is used to obtain the precipitated phase of Al-Cu-Mg aluminum alloy, as shown in Figure 8. Based on EDS analysis, the precipitates were tentatively identified as Al_2_Cu and Al_2_CuMg phases. There are small holes in some areas, which are caused by the precipitation falling off during the polishing process. The precipitate content of each sample is calculated using ImageJ software, and the results are shown in Table 7.

With the increasing annealing temperature, the precipitated phase content of the sample increases first and then decreases. There are some changes between the precipitated phases of the samples under annealing treatment. However, due to the low temperature, the solution effect is poor, and the precipitated phase content is concentrated in the middle of 4.2%~4.9%, and the change is small. With the increase in solution temperature and solution time, the precipitated phase content of the sample decreased gradually. This is because the precipitated phases Al_2_Cu and Al_2_CuMg, which are included around the aluminum matrix, gradually dissolve into the matrix with the increase in solution temperature and solution time, resulting in a decrease in the content of the precipitated phase. It can be found that the influence of solution temperature change on precipitated phase content is greater than that of solution time.

#### 3.2.3. Laser Ultrasonic Detection of Microstructure

**Figure 9:**
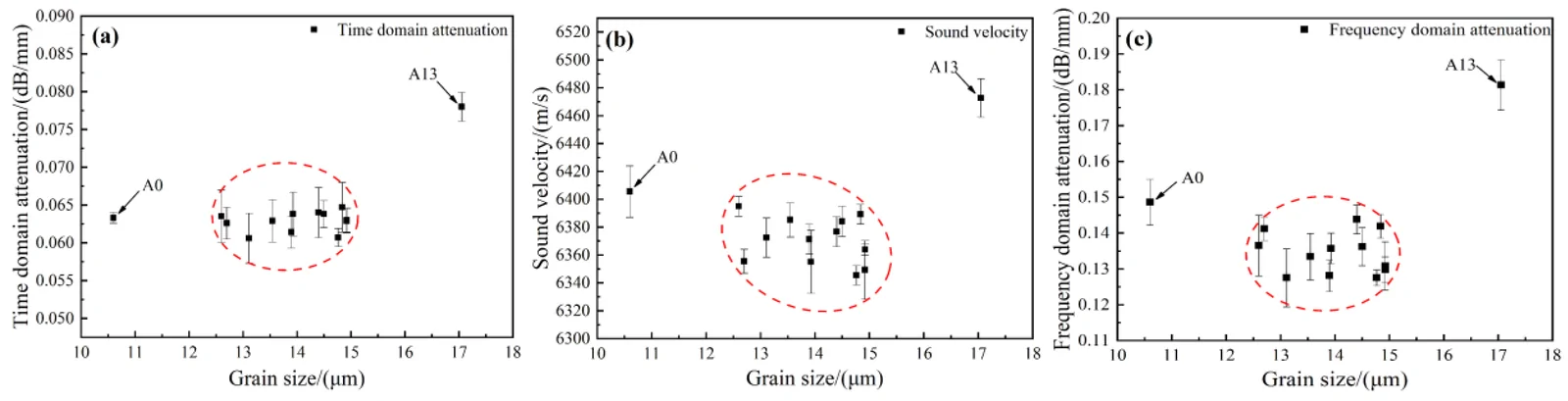
(**a**) Grain size and attenuation coefficient in frequency domain; (**b**) grain size and ultrasonic velocity; (**c**) grain size and attenuation coefficient in frequency domain. Error bars represent the standard deviation of three repeated ultrasonic measurements at different positions on each specimen.

The relationship between the attenuation coefficient in the time domain, velocity and attenuation coefficient in the frequency domain and precipitated phase content is analyzed. It is found that sample A13 is the deviation point of abrupt change in precipitated phase content and laser ultrasonic characteristic value. Among the specimens, sample A13 represents an extreme case, characterized by the coarsest grain size and the lowest precipitate content due to the most severe solution treatment (500 °C for 360 min). This unique microstructure results in a significant deviation of its ultrasonic characteristic values from the main cluster, providing a valuable endpoint for exploring the sensitivity of the laser ultrasonic technique. The average grain size of the sample is fitted and analyzed with the attenuation coefficient in the time domain, sound velocity and attenuation coefficient in the frequency domain, as shown in Figure 9.

By analyzing the ultrasonic characteristic value and microstructure of A0–A13 samples, it can be found that the grain size of A0 and A13 samples has significantly changed, and both of them are not in the range of 12.5~15 μm. The grain size of the A0 sample is much smaller than that of other samples, while that of the A13 sample is much larger than that of other samples. This is because the precipitation content around the aluminum matrix is reduced, the inhibition effect on grain growth is weakened, and the grain growth is caused by the increase in solution temperature and solution time. Only sample A13 exhibited a significant change in its ultrasonic characteristic values. In contrast, the values for sample A0 remained within the same range as the majority of the other specimens.

**Figure 10:**
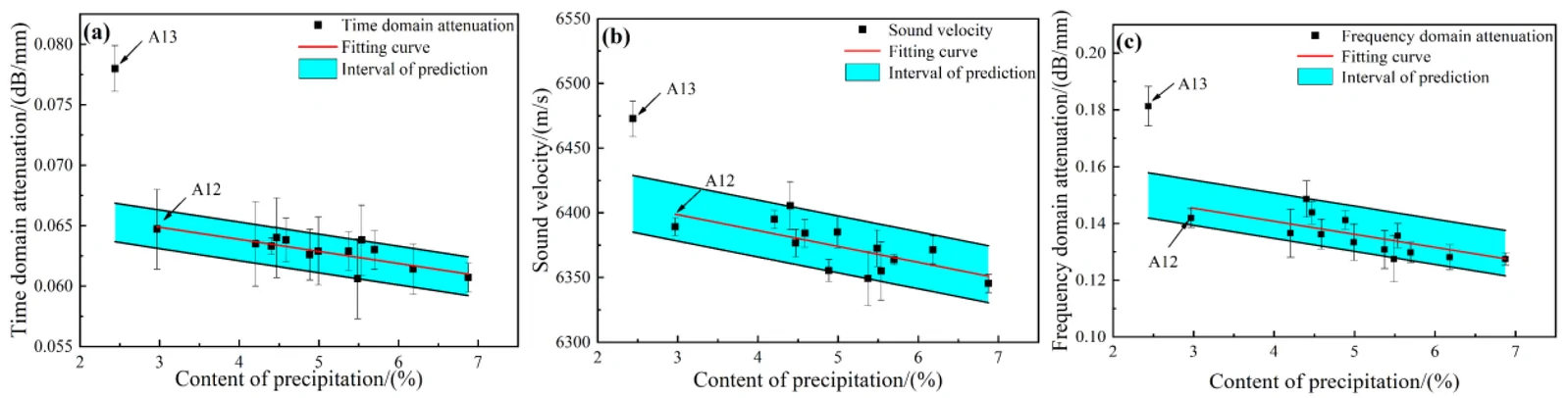
(**a**) Precipitated phase content and attenuation coefficient in time domain; (**b**) precipitated phase content and ultrasonic velocity; (**c**) precipitated phase content and attenuation coefficient in frequency domain. Error bars represent the standard deviation of three repeated ultrasonic measurements at different positions on each specimen.

In order to analyze the distribution of precipitated phase content and laser ultrasonic signal characteristic value of Al-Cu-Mg aluminum alloy, the precipitated phase content is coupled with the laser ultrasonic characteristic value, respectively (as shown in Figure 10).

The precipitated phase content of A12 and A13 samples is obviously smaller than that of other samples. Sample A13 deviates far from the predicted interval, and its laser ultrasonic signal characteristic value increases significantly. The precipitated phase content of the A12 sample increased, but the change amplitude of the laser ultrasonic signal characteristic value is small and could be ignored. Therefore, when only one of the precipitated phase content and average grain size of Al-Cu-Mg aluminum alloy deviates, the three ultrasonic characteristic values of laser ultrasonic attenuation coefficient in the time domain, frequency domain and sound velocity cannot deviate. The characteristic value of the laser ultrasonic signal will change significantly and deviate from other samples only when the precipitated phase content and average grain size of Al-Cu-Mg aluminum alloy change significantly.

#### 3.2.4. Laser Ultrasonic Detection of Mechanical Properties

T6 solution-aging heat treatment process has a significant effect on the optimization of the properties of Al-Cu-Mg aluminum alloy. The elongation L_e_ and tensile force F of the sample are obtained by tensile test. The tensile strength, yield strength and elongation of each Al-Cu-Mg aluminum alloy sample are calculated by substituting the data into the formula, as shown in Table 8. At the same time, the length of the diagonal of the indentation rectangle, the relative Angle of the indenter and the applied load are measured from the microhardness diagram. According to the micro-Vickers hardness formula, the Vickers hardness of the specimen surface is calculated. The average microhardness of each sample after 10 hardness tests is shown in Table 8.

It is found that the tensile strength, yield strength and microhardness of the samples are proportional to the solution temperature and solution time. The influence of solution temperature on the strength and microhardness is more significant than that of solution time. The content of the precipitated phase decreases with the increase in solution temperature and solution time. As a result, the hard precipitated phases Al_2_Cu and Al_2_CuMg, which were originally included around the aluminum alloy matrix dissolved into the matrix, gradually increased, and the solid solution strengthening of the matrix is improved. Thus, the strength and hardness of Al-Cu-Mg aluminum alloy are improved. Therefore, it is necessary to use laser ultrasonic detection technology to enable rapid characterization of the mechanical properties of Al-Cu-Mg aluminum alloy.

**Figure 11:**
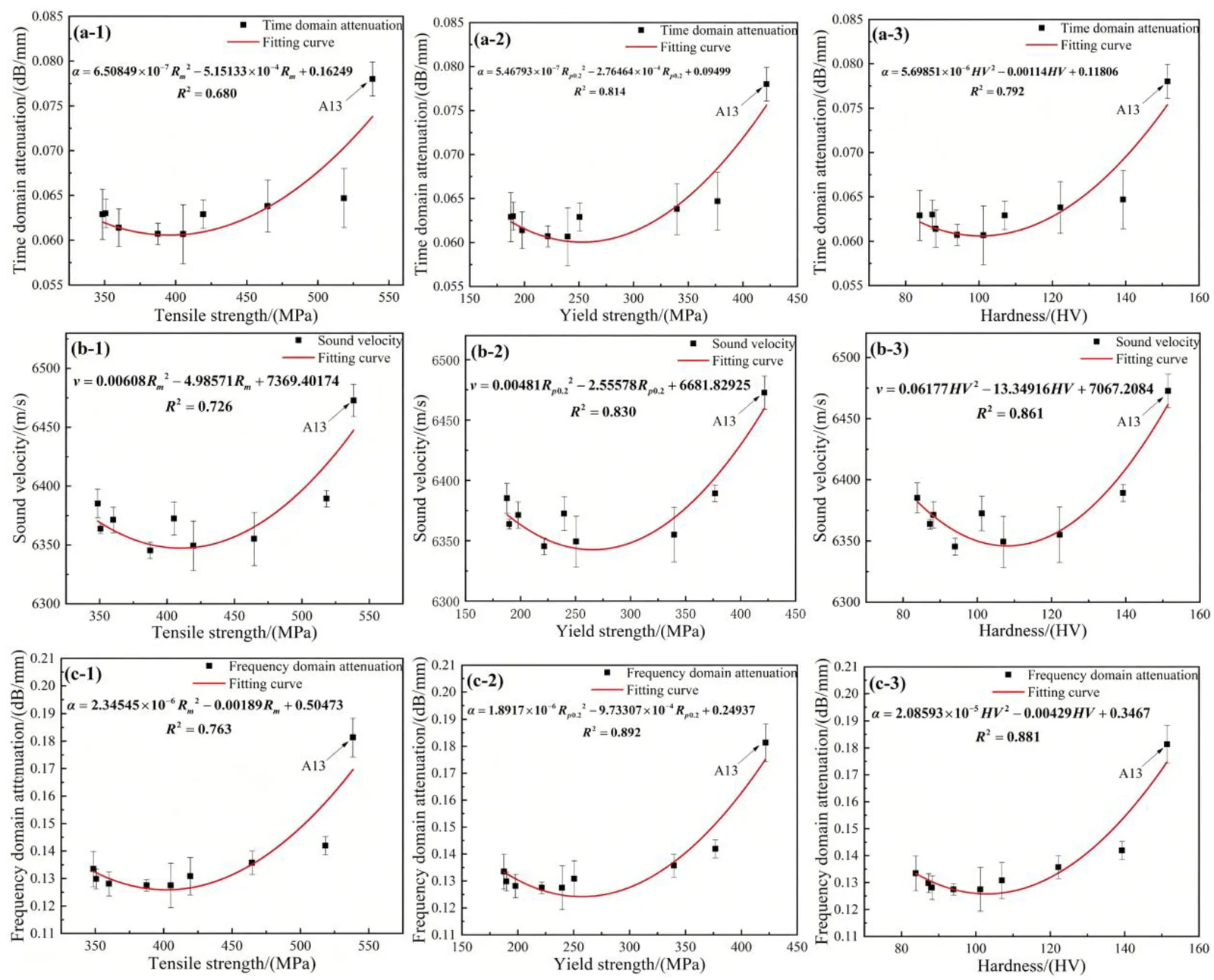
Fitting curve of mechanical properties and laser ultrasonic signal characteristic values: attenuation coefficient in time domain and (**a-1**) tensile strength, (**a-2**) yield strength, (**a-3**) microhardness; sound velocity and (**b-1**) tensile strength, (**b-2**) yield strength, (**b-3**) microhardness; attenuation coefficient in frequency domain and (**c-1**) tensile strength, (**c-2**) yield strength, (**c-3**) microhardness. Error bars represent the standard deviation of three repeated ultrasonic measurements at different positions on each specimen.

The optimal relationship curves between the eigenvalues of the laser ultrasonic signal and the three main mechanical properties of Al-Cu-Mg aluminum alloy samples, tensile strength, yield strength and microhardness, are obtained by using the least square method, as shown in Figure 11.

The yield strength and attenuation coefficients in the time domain, sound velocity and attenuation coefficients in the frequency domain are fitted by second-order polynomial. The goodness of fit R^2^ values are 0.81, 0.83 and 0.89, respectively (Figure 11(a-2), Figure 11(b-2) and Figure 11(c-2)). The second-order polynomial fitting between hardness and attenuation coefficient in the time domain, sound velocity and attenuation coefficient in the frequency domain is used, and a better fitting is also obtained. The obtained goodness of fit R^2^ values are 0.79, 0.86 and 0.88, respectively (Figure 11(a-3), Figure 11(b-3) and Figure 11(c-3)). However, the relationship between tensile strength and attenuation coefficient in the time domain, sound velocity and attenuation coefficient in the frequency domain is relatively weak (R^2^ are 0.68, 0.73 and 0.76).

The relationship goodness of the three eigenvalues and the three mechanical properties can be compared respectively. The quadratic polynomial fits, which include the extreme condition of sample A13, demonstrate that the ultrasonic parameters can effectively track the evolution of mechanical properties across a wide property range, from the relatively soft (e.g., A0) to the hardest condition (A13). In the coupling of the three mechanical properties, the attenuation coefficient in the frequency domain is greater than the attenuation coefficient in the time domain (*α_frequency domain_* > v > *α_Time_*). This indicates that the three ultrasonic characteristic values can characterize the mechanical properties of Al-Cu-Mg aluminum alloy, and there is a good correlation between the yield strength and microhardness. These empirical correlations indicate that, within the present dataset, the ultrasonic characteristic values exhibit reasonable consistency with the measured yield strength and microhardness. Moreover, the relationship goodness of the attenuation coefficient in the frequency domain is slightly better than the two eigenvalues in the time domain, suggesting that in this experimental context, the frequency-domain attenuation coefficient may offer a more promising indicator for characterizing the mechanical properties of Al-Cu-Mg aluminum alloy.

Interpreting the Polynomial Correlations Mechanistically: Higher solution temperature and prolonged holding time dissolve Al_2_Cu/Al_2_CuMg precipitates into the matrix, enhancing solid solution strengthening and thereby elevating strength and hardness. Concurrently, the reduced precipitate content weakens phase-boundary scattering, whereas grain coarsening intensifies grain boundary scattering. The net scattering response, dominated by grain boundary effects under the present conditions, manifests most sensitively in the frequency-domain attenuation coefficient, explaining its superior fitting performance. These coupled scattering and solid solution hardening effects underpin the observed polynomial trends; however, fully quantitative decoupling grain size and precipitate contributions would require additional controlled experiments with broader microstructural variations.

### 3.3. Evaluation Scheme of Microstructure and Mechanical Properties of Aluminum Alloy Based on Laser Ultrasonics

**Figure 12:**
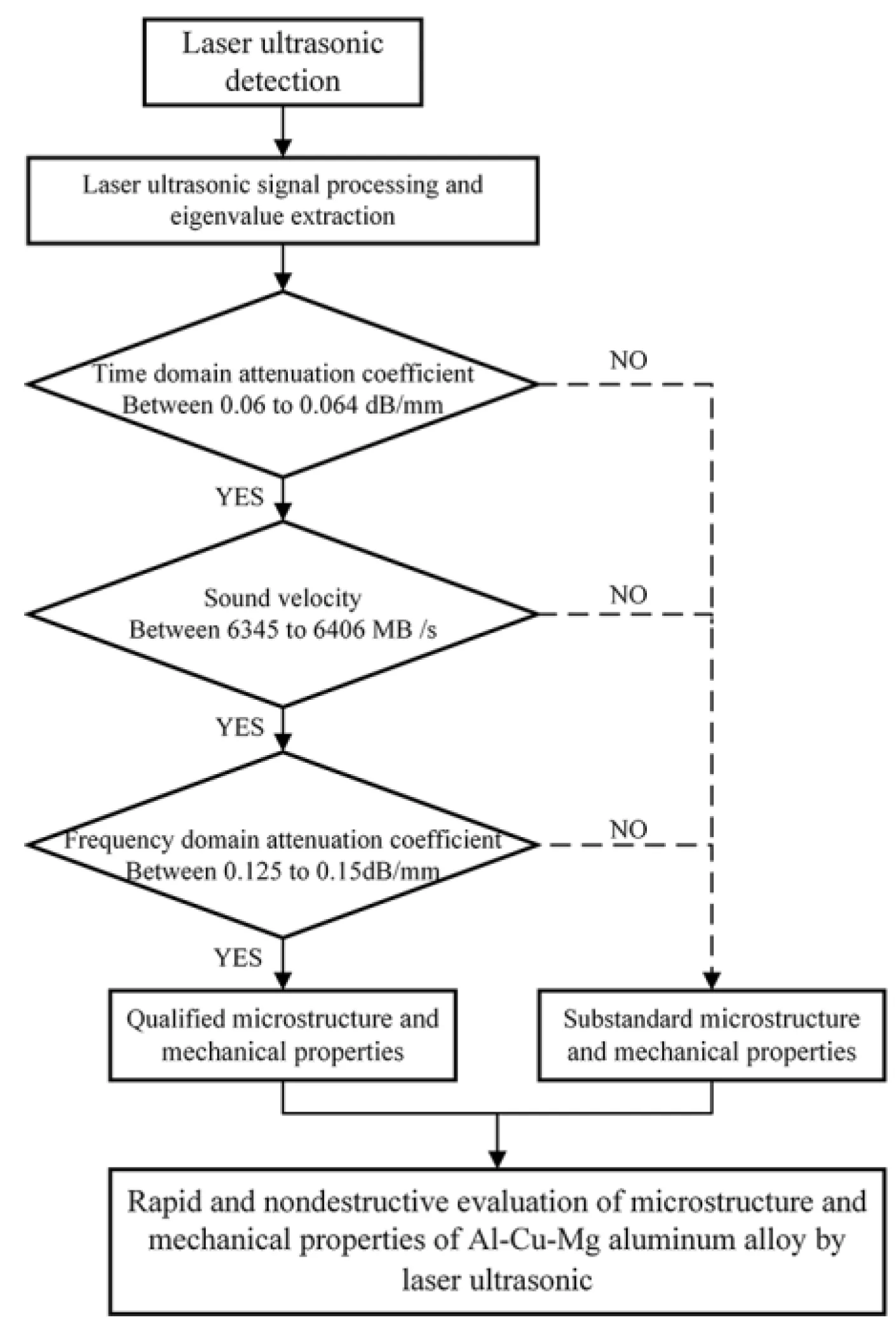
Flow chart of microstructure and mechanical properties evaluation of aluminum alloy.

In this paper, the microstructure and mechanical properties of Al-Cu-Mg aluminum alloy are examined by comparing the laser ultrasonic signal characteristic values with predefined reference ranges. When the attenuation coefficient in the time domain, sound velocity and attenuation coefficient in the frequency domain of laser ultrasonic detection of Al-Cu-Mg aluminum alloy are not within the ranges of 0.06~0.064 dB/mm, 6345~6406 m/s and 0.125~0.15 dB/mm, respectively, it may be preliminarily identified that the microstructure and mechanical properties of the sample have deviated from those of the typical T6-treated specimens. The specific flow chart is shown in Figure 12. This offers a potential approach for rapid screening of Al-Cu-Mg aluminum alloy samples within the current dataset, as the microstructure and mechanical properties may be preliminarily assessed by examining whether the ultrasonic characteristic values fall within the empirically observed intervals.

It should be noted that these reference ranges are empirically derived from specimens A0–A12 (with sample A13 excluded from the correlation analysis due to its significant deviation from the general trend) and are intended as preliminary reference values rather than universally applicable qualification standards. Therefore, the proposed empirical correlations and reference intervals are strictly applicable only within the microstructural range represented by specimens A0–A12; their validity for microstructures substantially outside this range, such as that of sample A13, requires further independent validation. In practical engineering applications, the corresponding ranges should be re-established according to the allowable tolerances of actual production requirements and material specifications. Furthermore, samples identified as falling outside these ranges require additional verification using conventional destructive testing methods. The proposed approach is complementary to, rather than a substitute for, established characterization techniques. The consistency between the laser ultrasonic observations and the microstructural influences on mechanical properties supports the plausibility of this method, while its general applicability and predictive performance require further validation using independent test samples and a broader range of processing conditions.

## 4. Conclusions

In this paper, Al-Cu-Mg aluminum alloy is taken as the research object. Through the metallographic experiment, EBSD experiment, tensile test and microhardness test, and laser ultrasonic detection technology and VMD signal noise reduction method, the following conclusions are drawn:

(1) The attenuation coefficient and sound velocity of laser ultrasonic signal are inversely proportional to the precipitated phase content. When the precipitated phase content of the sample changes, the attenuation and sound velocity of the sample also change, deviating from the general trend observed in the majority of specimens.

(2) When the eigenvalues are coupled with the three mechanical properties, frequency-domain attenuation is better than sound velocity than time domain (*α_frequency domain_* > v > *α_Time_*). The ultrasonic characteristic values show relatively stronger correlations with the yield strength and microhardness.

(3) For the Al-Cu-Mg aluminum alloy specimens investigated in this study, the observed ranges of time-domain attenuation coefficient, ultrasonic velocity, and frequency-domain attenuation coefficient are reported as empirical observations for the specific heat-treated conditions. These ranges are not intended as validated qualification criteria but may serve as preliminary reference values for comparison.

By comparing the laser ultrasonic non-destructive testing results with these empirically derived numerical ranges, preliminary identification of whether the microstructure and mechanical properties of Al-Cu-Mg aluminum alloy have deviated from the typical condition represented by specimens A0–A12 can be made. However, it must be emphasized that these correlations and reference intervals were established based on specimens A0–A12 (excluding sample A13) and are therefore strictly applicable only within the investigated microstructural range. Their applicability to microstructures significantly outside this range, or to other alloy systems or processing conditions, requires further validation through independent experiments. These findings suggest the potential of laser ultrasonic detection for characterizing the microstructure and mechanical properties of Al-Cu-Mg aluminum alloy and offer a preliminary reference for non-contact rapid evaluation within the tested parameter space.

## Figures and Tables

**Table 1:** The chemical composition of Al-Cu-Mg aluminum alloy [23].

Chemical	Cu	Mg	Mn	Fe	Zn	Si	Ti	Al
Content (%)	3.35	1.70	0.61	0.14	0.11	0.07	0.04	Bal.

**Table 2:** The heat treatment process of Al-Cu-Mg aluminum alloy.

No.	Temp. (°C)	Time (min)	Cooling	Temp. (°C)	Time (min)	Cooling
A0	\	\	\			
A1	200	120	FC			
A2	250	120	FC			
A3	300	120	FC			
A4	350	120	FC			
A5	400	120	WC	180	720	AC
A6	400	240	WC			
A7	400	360	WC			
A8	450	120	WC			
A9	450	240	WC			
A10	450	360	WC			
A11	500	120	WC			
A12	500	240	WC			
A13	500	360	WC			

FC is furnace cooling, WC is water cooling, and AC is air cooling.

**Table 3:** Thickness of Al-Cu-Mg aluminum alloy samples.

No.	Average Thickness (mm)	No.	Average Thickness (mm)
A0	1.83	A7	1.88
A1	1.87	A8	1.89
A2	1.87	A9	1.90
A3	1.90	A10	1.89
A4	1.88	A11	1.88
A5	1.89	A12	1.91
A6	1.89	A13	1.85

**Table 4:** Characteristic value of laser ultrasonic signal of Al-Cu-Mg aluminum alloy.

No.	*α_Time_* (dB/mm)	*V* (m/s)	*α_Frequency_* (dB/mm)	Coupling Error
A0	0.063	6406	0.149	0.006
A1	0.063	6356	0.141	0.003
A2	0.064	6384	0.136	0.005
A3	0.064	6377	0.144	0.004
A4	0.064	6395	0.137	0.008
A5	0.063	6385	0.133	0.006
A6	0.061	6371	0.128	0.004
A7	0.063	6364	0.130	0.004
A8	0.061	6345	0.128	0.002
A9	0.061	6373	0.128	0.008
A10	0.063	6349	0.131	0.007
A11	0.064	6355	0.136	0.004
A12	0.065	6389	0.142	0.003
A13	0.078	6473	0.181	0.007

*α_Time_* is the attenuation coefficient in the time domain, *V* is ultrasonic velocity, and *α_Frequency_* is the attenuation coefficient in the frequency domain.

**Table 5:** Average grain size of the sample.

No.	Average Grain Size D (μm)	No.	Average Grain Size D (μm)
A0	10.6	A7	14.9
A1	12.7	A8	14.8
A2	14.5	A9	13.1
A3	14.4	A10	14.9
A4	12.6	A11	13.9
A5	13.5	A12	14.8
A6	13.9	A13	17.1

**Table 6:** Grain size features of Al-Cu-Mg aluminum alloy.

Sample No.	Turner Grain Size ($\tilde{D}$)	Standard Deviation (σ)	Expected Value (μ)	Sample No.	Turner Grain Size ($\tilde{D}$)	Standard Deviation (σ)	Expected Value (μ)
A0	11.0	0.73	2.13	A7	15.2	0.77	2.43
A1	12.8	0.62	2.36	A8	15.0	0.76	2.42
A2	15.8	0.78	2.46	A9	13.5	0.78	2.30
A3	16.4	0.90	2.40	A10	15.3	0.75	2.45
A4	13.4	0.77	2.30	A11	14.7	0.65	2.48
A5	14.2	0.75	2.37	A12	15.4	0.82	2.40
A6	16.1	0.88	2.39	A13	17.0	0.78	2.53

**Table 7:** Precipitate contents.

Sample No.	Precipitated Phase Content (%)	Sample No.	Precipitated Phase Content (%)
A0	4.4	A7	5.7
A1	4.9	A8	6.9
A2	4.6	A9	5.5
A3	4.5	A10	5.4
A4	4.2	A11	5.5
A5	5.0	A12	3.0
A6	6.2	A13	2.4

**Table 8:** Mechanical properties of Al-Cu-Mg aluminum alloy.

No.	Tensile Strength R_m_ (MPa)	Yield Strength R_p0.2_ (MPa)	Rate of Elongation δ (%)	Average Hardness of Sample (HV)
A5	348.5 ± 3.6	187.7 ± 2.0	12.6 ± 1.0	83.8 ± 0.7
A6	360.1 ± 2.9	198.0 ± 4.1	15.7 ± 1.3	88.2 ± 0.7
A7	350.7 ± 3.0	189.9 ± 4.8	17.7 ± 1.3	87.3 ± 0.5
A8	387.5 ± 8.7	221.6 ± 8.8	15.1 ± 0.7	94.0 ± 1.0
A9	405.2 ± 6.9	239.7 ± 7.2	17.0 ± 1.9	101.2 ± 0.6
A10	419.5 ± 3.0	250.6 ± 4.9	19.3 ± 0.8	107.0 ± 1.3
A11	464.7 ± 4.1	339.6 ± 5.3	13.0 ± 0.6	122.2 ± 0.9
A12	518.4 ± 4.7	376.7 ± 4.5	17.1 ± 2.6	139.3 ± 2.2
A13	538.5 ± 5.3	421.7 ± 7.4	13.5 ± 1.3	151.4 ± 2.9

## Data Availability

The original contributions presented in this study are included in the article. Further inquiries can be directed to the corresponding author.
